# Anti-Diabetic, Anti-Oxidant and Anti-Hyperlipidemic Activities of Flavonoids from Corn Silk on STZ-Induced Diabetic Mice

**DOI:** 10.3390/molecules21010007

**Published:** 2015-12-23

**Authors:** Yan Zhang, Liying Wu, Zhongsu Ma, Jia Cheng, Jingbo Liu

**Affiliations:** College of Food Science and Engineering, Jilin University, Changchun 130062, China; zy01@jlu.edu.cn (Y.Z.); wuliying-lisa@hotmail.com (L.W.); zsma@jlu.edu.cn (Z.M.); chengj_369@sohu.com (J.C.)

**Keywords:** anti-diabetic, anti-oxidant, anti-hyperlipidemic, corn silk, flavonoids

## Abstract

Corn silk is a well-known ingredient frequently used in traditional Chinese herbal medicines. This study was designed to evaluate the anti-diabetic, anti-oxidant and anti-hyperlipidemic activities of crude flavonoids extracted from corn silk (CSFs) on streptozotocin (STZ)-induced diabetic mice. The results revealed that treatment with 300 mg/kg or 500 mg/kg of CSFs significantly reduced the body weight loss, water consumption, and especially the blood glucose (BG) concentration of diabetic mice, which indicated their potential anti-diabetic activities. Serum total superoxide dismutase (SOD) and malondialdehyde (MDA) assays were also performed to evaluate the anti-oxidant effects. Besides, several serum lipid values including total cholesterol (TC), triacylglycerol (TG), low density lipoprotein cholesterol (LDL-C) were reduced and the high density lipoprotein cholesterol level (HDL-C) was increased. The anti-diabetic, anti-oxidant and anti-hyperlipidemic effect of the CSFs suggest a potential therapeutic treatment for diabetic conditions.

## 1. Introduction

Diabetes mellitus [DM] is a serious chronic metabolic complication that results from abnormal insulin production or metabolism and chronic hyperglycemia [1]. DM is characterized by carbohydrate, lipid and protein metabolism disturbances and hyperglycemia, as well as oxidative stress accompanied with the main clinical symptoms polydipsia, polyuria, polyphagia, high urine glucose level and weight loss [2,3]. It reported that 7% of the adults around the world suffer from DM [4]. Recently, there has been a sharp increase in DM levels which parallels that of obesity and overweight. It projected by the International Diabetes Federation that by the year 2030 the number of diabetic patients will be approximately 552 million [5]. There are two major classes of DM, which are Type 1 DM (T1DM) and Type 2 DM (T2DM) [6]. Of the two types, T2DM cases are prevalent, with only 5%–10% corresponding to T1DM [7].

At present, insulin and oral anti-diabetic chemical agents (*i.e.*, glucosidase inhibitor, biguanides, insulin sensitizer and sulfonylureas, *etc.*) are used in clinical practice as therapies for DM [8]. Many many of them have some limitations and side effects, such as liver and kidney failure, hypoglycemia, diarrhea and lactic acidosis which are difficult to tolerate [9,10]. Therefore, in order to protect patients from these negative effects of synthetic agents, the search for new compounds with better effectiveness and lower toxicity has received more and more attention as a potential source of new therapeutic anti-diabetic drugs for DM patients. This has encouraged investigation searching for alternative remedies derived from traditional herbal medicines which are accepted as valuable resources for primary healthcare by the World Health Organization (WHO) [11,12].

Corn is one of the top three most widely cultivated cereal crops in the world. Corn silk (*Zea mays* L.) is the style and stigma of corn fruit and is a waste material from corn cultivation and thus available in abundance throughout the world [13]. Corn silk is known as a traditional Chinese herbal medicine which has been widely used to treat edema, cystitis, gout, nephritis, kidney stones, obesity, as well as prostatitis and similar ailments [14]. It also reported that corn silk possesses hypoglycemic, anti-tumor, antioxidant, anti-fatigue and anti-fungal properties [15]. Meanwhile, corn silk contains various chemical components including polysaccharides, proteins, flavonoids, vitamins, minerals, alkaloids and tannins, as well as steroids, *etc.* [16]. Previous studies have showed that among all the components, flavonoids can be regarded as the main contributors to most of the therapeutic effects, including anti-oxidant, anti-aging, diuretic, and anti-proliferative activity on human cancer cell lines, *etc*. [15]. As mentioned, oxidative stress, as well as lipid metabolism disturbances play an important role in diabetes besides hyperglycemia, hence, drugs with several properties would be much more effective in the treatment of diabetes [17]. However, data regarding the corn silk flavonoids’ *in vivo* anti-diabetic, anti-oxidant and anti-hyperlipidemic activities are very limited, with only a few studies performed that demonstrated their anti-oxidant capacity. Flavonoids from some rare or regionally limited natural plant materials, such as *Sanguis draxonis* [18], *Malus toringoides* (Rehd.) Hughes leaves [19], and *Pilea microphylla* (L.) [20] were proved to possess anti-diabetic activity, which implies that there is a good chance that flavonoids from corn silk also have anti-diabetes capability.

In this regard, it made great sense to evaluate the anti-diabetic, anti-oxidant and anti-hyperlipidemic activities of flavonoids from corn silk in a STZ-induced diabetic mice model to identify a more abundant natural source for discovering new DM therapies which might be more effective with less side effects and readily accessible to all the diabetic population.

## 2. Results and Discussion

### 2.1. Total Flavonoids and Total Phenolic Content

Previous studies reported that the total phenolic and the total flavonoids content of its extracts were associated with the pharmacological effects of corn silk, such as the antioxidant, anti-inflammatory, and antioxidant activities or diuretic activity [21,22]. Hence, in this study, ethanol was used as our extraction solvent to obtain the crude corn silk extract. The total phenolic content of the corn silk extracts was determined through a linear gallic acid standard curve (y = 0.1896x + 0.4326; *R*^2^ = 0.9984) and the total phenolic content was 34.6 ± 0.2 milligram of gallic acid equivalents per gram of corn silk extract. The total flavonoids content of the corn silk extracts was evaluated by an aluminium chloride colorimetric assay, using rutin as a standard (y = 28.42x − 0.008, *R*^2^ = 0.9991) and the total flavonoids content was 16.8 ± 0.4 milligram of rutin equivalents per gram of corn silk extract.

### 2.2. Effect of CSFs on Body Weight of Normal and STZ-Induced Diabetic Mice

**Figure 1:**
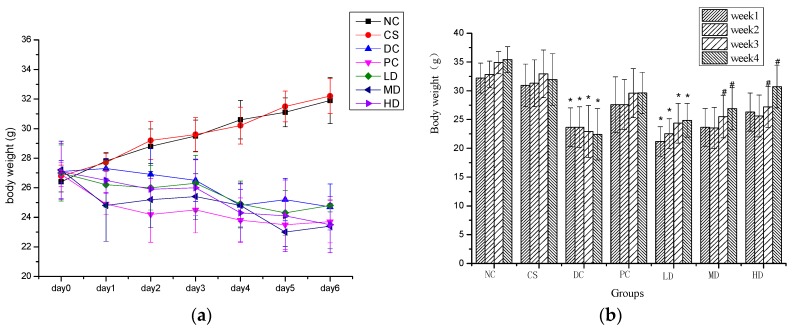
(**a**) Body weight of normal and STZ-induced diabetic mice in first 6 days after the STZ treatment; (**b**) Body weight of normal and STZ-induced diabetic mice in four weeks. Results were presented as means ± SD (*n* = 10). The columns of each index in have * *p* < 0.05, *vs.* NC; # *p* < 0.05, *vs.* week 1. NC, CS, DC, PC, LD, MD and HD are abbreviations for non-diabetic control group, non-diabetic CSFs high dose group, diabetic control group, diabetic CSFs low dose group, diabetic CSFs medium dose group, and diabetic CSFs high dose group, respectively.

An earlier investigation has demonstrated the anti-oxidant and free radicals scavenging activity [23] of the components of corn silk flavonoids. In the present study, STZ rats showed slight anti-hyperglycemic and anti-hyperlipidemic activity. The acute effect of STZ-Induced T2DM model (MI) on body weight was determined by measuring the body weights of mice in the week following the MI process. Body weights of mice in the diabetic control (DC) group were significantly decreased by 7 days after the STZ treatment, while that of the non-diabetic control (NC) animals were significantly elevated with a statistical significance level at *p* < 0.01. Figure 1a shows that during the first two days, there was no obvious difference on body weights amongst all seven groups and on day 3, the situation had changed, with no significant difference noted between the two NC groups and among the DC groups, while body weights of the NCs significantly differed from those of the DC groups.

Weight loss is one of the most important symptoms of DM, so we observed the body weights of mice by measuring them weekly. As shown in Figure 1b, when compared to NCs, no significant difference was found between the NC and CS groups, which implied that CSFs did not have any obvious effect on the body weight of normal mice, while some groups, including the DC and LD groups, showed an opposite result. A constant weight loss was noted in the DC group during the whole experimental period, while mice in both the PC and the groups administrated with CSFs gained weight during the four weeks. This suggested that CSFs and dimethylbiguanide can minimize the body weight loss of DM mice to a different extent. The effect of dimethylbiguanide on weight loss of DM mice was better than that of CSFs and the effect of CSFs was positively dose correlated through no significant diffidence was observed. The weight loss of STZ-induced diabetic mice is a symptom of diabetes, which is in agreement with the reported anti-diabetic activity of embelin in STZ-treated rats [17].

### 2.3. Effect of MI and CSFs on Fasting BG of Normal and STZ-Induced Diabetic Mice

**Figure 2:**
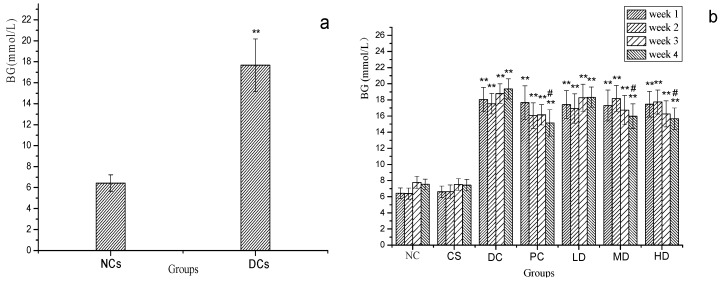
(**a**) Fasted BG levels of NCs [normal mice] and DCs [streptozotocin-induced diabetic mice] on the sixth day; (**b**) Fasted BG levels of NC, CS, DC, PC, LD, MD and HD in four weeks. Results were presented as means ± SD (*n* = 10). The columns of each index in Figure 2a have ** *p* < 0.01 *vs.* NCs. The columns of each index in Figure 2b have ** *p* < 0.01 *vs.* NC; # *p* < 0.05 *vs.* week1. Group abreviations are as given in the caption of Figure 1.

Figure 2a displays the fasting BG level of mice on the sixth day after the MI. According to Figure 2a the fasting BG level of DC mice were 17.67 mmol/L which was significantly (*p* < 0.01) higher than that of NCs and above the afore mentioned standardized DM value, 11.1 mmol/L, so that we considered the diabetes induction succeeded.

Figure 2b reveals the alteration of BG concentration in different groups correlated with the duration of the experiments. BG values of mice in NCs remained almost unchanged during the four weeks, which differed significantly from that of DCs with a statistical significance level at *p* < 0.01. BG values of DC and LD were continuously rising, which indicated that low dose (100 mg/kg) CSFs had no observable effect on lowering the BG level of DM mice, while BG of PC decreased after being treated with dimethylbiguanide and BG level of MD and HD decreased in week 3 and week 4, but the BG value of these three groups did not recover to the normal level at the end of this experiment (*p* < 0.05). This suggested that dimethylbiguanide and the administration of 300 mg/kg and 500 mg/kg CSFs can lower the BG concentration of diabetic mice. This observation was consistent with previous reports about otherphy to chemicals [24,25].

### 2.4. Effect of CSFs on Water Consumption and Food Intake of Normal and STZ-Induced Diabetic Mice

**Figure 3:**
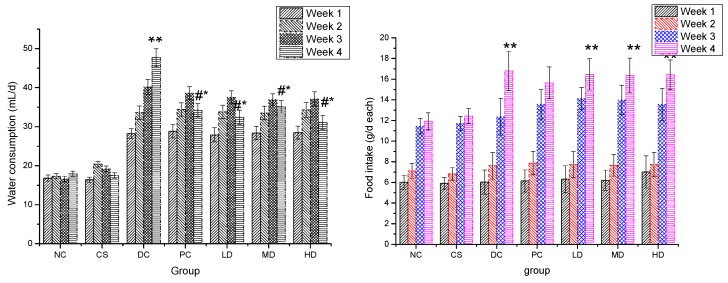
Water consumption (**a**) and food intake (**b**) of normal and STZ-induced diabetic mice. Results are presented as means ± SD (*n* = 10). The columns of each index have * *p* < 0.05, ** *p* < 0.01 *vs.* NC; # *p* < 0.05 *vs.* week 1. Group abbreviations are as given in the caption of Figure 1.

The effect of CSFs on daily water consumption and food intake of mice is shown in Figure 3. Water consumption of NC mice increased continuously and slightly during the 4 weeks, but the rise was not significant with water consumption remaining between 16.6 to 20.4 mL/day each. Both water intake and urinary output of DCs on the other hand were significantly elevated. The maximum water consumption increase was noted in the DC group which went from 28.2 to 47.7 mL/day each, while, the cage of DC animals got very wet, which was in accordance with the typical symptoms of diabetic polydipsia and polyuria. According to Figure 3a, the water consumptions of both the PC and the groups which received CSFs were all decreased in the fourth week when compared with that in the first three weeks, which indicated that the polydipsia and polyuria symptoms were improved to a certain degree. The water intake values of the PC and groups treated with CSFs were significantly lower than that of DC (*p* < 0.05) which suggested that both dimethylbiguanide and CSFs can effectively inhibit the increase of water consumption. No significant differences were found between the NC and CS groups. As shown in Figure 3b, food consumptions in all groups were elevated as time elapsed with that of NCs’s and PC’s increasing more slowly, though there was no significant variation observed among the food intakes of the seven groups during the four-week experimental period.

### 2.5. Effect of CSFs on Related Organ Weight and Liver Glycogen of Normal and STZ-Induced Diabetic Mice

**Figure 4:**
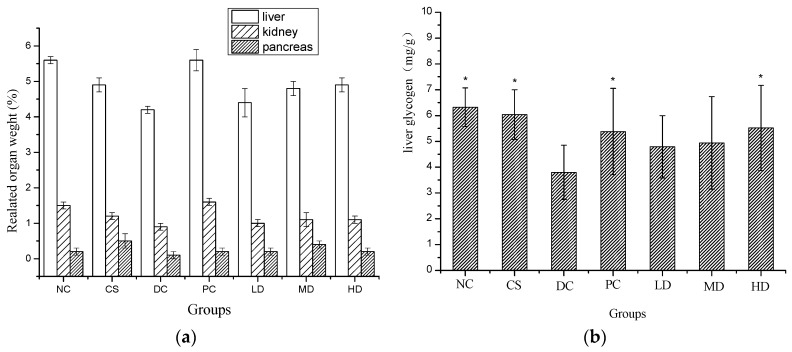
Related organ weight (**a**) and liver glycogen (**b**) of normal and STZ induced diabetic mice. Results were presented as means ± SD (*n* = 10). The columns of each index have * *p* < 0.05 *vs.* NC; Relative liver weight (%) = absolute kidney weight (g)/final body weight (g); relative kidney weight (%) = absolute kidney weight (g)/final body weight (g); relative pancreas weight (%) = absolute pancreas weight (g)/final body weight (g). Group abbreviations are as given in the caption of Figure 1.

As shown in Figure 4a, STZ led to the damage of the liver, kidney, and pancreas. Livers of mice in the DC group were much more damaged than in any other group (*p* < 0.05) and the pancreases of DC mice were also seriously damaged. When compared to DC, no significant damage was observed in kidneys in both the NC and PC groups and in pancreases in the CS and HD groups (*p* < 0.05). The related liver and kidney weights were elevated in the PC and HD groups, which indicated that both dimethylbiguanide and CSFs at high concentration can protect diabetic mice from liver and kidney damage.

The effect of CSFs on liver glycogen of mice is shown in Figure 4b. Liver glycogen levels in the NC, CS, PC, as well as HD groups significantly differed from those of the DC, LD, and MD (*p* < 0.05) groups, with NC showing the highest and DC the lowest and values between NC and CS were close to each other and no significant difference was observed, which suggested that the ingestion of 500 mg/kg CSFs not only had no adverse effect on liver glycogen metabolism but could also prevent the liver glycogen of diabetic mice from decreasing.

### 2.6. Effect of CSFs on Serum SOD and MDA of Normal and STZ-Induced Diabetic Mice

**Figure 5:**
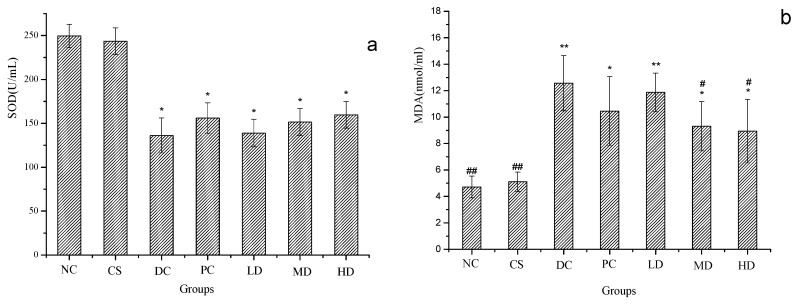
SOD (**a**) and MDA (**b**) of normal and STZ induced diabetic mice. Results were presented as means ± SD (*n* = 10). The columns of each index have * *p* < 0.05, ** *p* < 0.01 *vs.* NC; # *p* < 0.05, ## *p* < 0.01 *vs.* DC. Group abbreviations are as given in the caption of Figure 1.

Shown in Figure 5 are the SOD and MDA data, with Figure 5a showing the SOD value and Figure 5b the MDA value, respectively. When compared to NC, the SOD values of all the DC groups were significantly lower at *p* < 0.05 and the MDA values of the DC groups were observed to display the opposite trend, while both those of CS had almost the same value as the NC group, which revealed that the anti-oxidant capacity of diabetic mice was damaged. 

No significant difference was found between the SOD value of the DC group (*p* < 0.05) and the serum SOD level was rising slightly with the higher concentration of CSFs. The level of SOD increased due to the production of superoxide, which has been implicated in cell dysfunction [24]. When compared to DC, the MDA values of PC, MD, and HD were significantly reduced (*p* < 0.05). It suggested that dimethylbiguanide and CSFs were able to repair the anti-oxidant capacity of diabetic mice, but the effects were not significant.

### 2.7. Effect of CSFs on Serum TC, TG HDL-C and LDL-C of Normal and STZ-Induced Diabetic Mice

**Figure 6:**
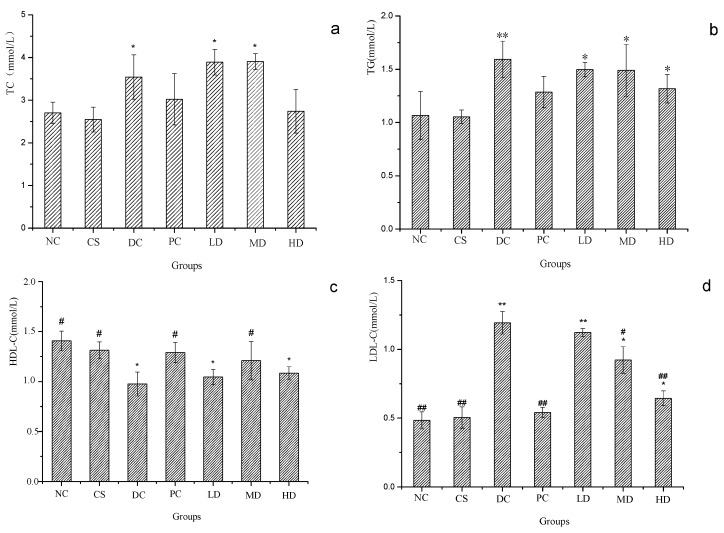
TC (**a**); TG (**b**); HDL-C (**c**) and LDL-C (**d**) of normal and STZ induced diabetic mice. Results were presented as means ± SD (*n* = 10). The columns of each index have * *p* < 0.05, ** *p* < 0.01 *vs.* NC; # *p* < 0.05, ## *p* < 0.01 *vs.* DC. Group abbreviations are as given in the caption of Figure 1.

Figure 6 displays the effect of CSFs on serum TC, TG, HDL-C and LDL-C levels of normal and streptozotocin-induced diabetic mice. The serum TC value obtained from the experiment is shown in Figure 6a. Compared to NC, significant variances were found in DC, LD, and MD groups with NC showing a lowest value (*p* < 0.05), and TC concentrations of diabetic mice treated with dimethylbiguanide and 500 mg/kg CSFs were also significantly lower than that of DC and close to the normal level at *p* < 0.05. Figure 6b displays the effect of CSFs on serum TG of mice. As shown, the TG value of DC animals were significantly increased when compared to NC at the significant level *p* < 0.01. When compared to DC, TG concentrations of mice in the PC group and mice administrated with CSFs obviously decreased, but it did not reach the normal value (*p* < 0.05).

The TG value in the HD group was similar to that of the PC one, all of which decreased and were the lowest values among the DC groups. This suggested that the ingestion of both 140 mg/kg dimethylbiguanide and 500 mg/kg CSFs was able to lower the serum TC and TG levels of diabetic mice to a similar extent. Serum HDL-C and LDL-C of mice are shown in Figure 6c,d, respectively. Compared to NC, HDL-C values of DC, LD, and HD were significantly lowered (*p* < 0.05), and that of NC, CS, PC, and MD were obviously higher than that of DC, *p* < 0.05. Besides, the HDL-C concentration of MD has no significance difference with that of PC, which indicated that 300 mg/kg CSFs increased the serum HDL-C as effectively as dimethylbiguanide did. As shown in Figure 6d, the LDL-C levels of DC and LD were significantly elevated when compared with that of NC with a significance level at 0.01. The LDL-C concentrations of MD and HD were lower than that in DC and LD, but higher than the value of NC and significant differences were observed between every two groups among these five groups (*p* < 0.05). Compared to DC, significant decreases of LDL-C concentration were noted in NC, CS, PC, and HD at *p* < 0.01 and in MD at *p* < 0.05 and the decreases were positively correlated with the CSFs concentration. As Figure 6 reveals, no apparent difference was found between PC and HD, which implied that CSFs were capable of reducing serum LDL-C and regulating the lipid metabolism of diabetic mice.

## 3. Experimental Section

### 3.1. Materials and Chemicals

Corn silk (958 species of Zheng Dan, collected in early October 2013) was obtained from Tianjing food company of Jilin Province (China), dried at 40 °C, ground and stored in a dry environment. Streptozotocin, citrate sodium and citric acid were obtained from Sigma Chemical Co. (St. Louis, MO, USA). Dimethyl biguanide was obtained from Feihong Drug Co. (Nanchang, China). Total cholesterol (TC), triacylglycerol (TG), high density lipoprotein cholesterol (HDL-C), and low density lipoprotein cholesterol (LDL-C), superoxide dismutase (SOD), malondialdehyde (MDA), as well as liver glycogen analytical kits were purchased from Shanghai Yuanmu Bioengineering Co. Ltd. (Shanghai, China). All the other reagents used in the investigation were of analytical grade.

### 3.2. Preparation of Corn Silk Flavonoids (CSFs)

Five kilograms of corn silk was cut into small pieces and ground, then extracted with 20 L of ethanol (80% (*v/v*)) in a rotary shaker at 60 °C for 3.5 h and thereafter filtered immediately. The corn silk residue was subjected to the aforementioned process in triplicate to extract more flavonoid components. The filtrates were concentrated to 2.5 L under low pressure and filtered again, then the enriched filtrates were freeze-dried and kept at 0–4 °C for the further study.

### 3.3. Determination of Total Phenolic Content

The contents of total phenolics in samples were analyzed by the Folin-Ciocalteu colorimetric method described previously [26,27], using gallic acid as a standard. Briefly, the appropriate dilutions of extracts were oxidized with Folin-Ciocalteu reagent and the reaction was neutralized with sodium carbonate. The absorbance of the resulting blue color was measured at 760 nm after 90 min by an ultraviolet-visible spectrophotometer (UV-2550, Shimadzu Corporation, Kyoto, Japan). The total phenolic content was determined using the standard gallic acid calibration curve and results were expressed as milligram gallic acid equivalents per gram dry mass of corn silk.

### 3.4. Determination of Total flavonoid Content

The contents of total flavonoid in samples were analyzed by the modified colourimetric aluminium chloride method [28]. In brief, a dilute solution of the extracts in methanol was mixed with 0.01 mol/L aluminium chloride in methanol. Then the mixture was allowed to stand for 10 min at room temperature. The absorbance of the reaction mixture was measured at 400 nm with an ultraviolet visible spectro photometer (UV-2550, Shimadzu Corporation). Again the blank consisted of all reagents and solvents, but without the sample. The total flavonoids content was determined using the rutin calibration curve, at concentrations from 0.005 to 0.125 mg/mL in methanol, and expressed as milligram of rutin equivalents per gram dry mass of corn silk.

### 3.5. Animals and Diets

Seventy male mice weighted from 18 g to 20 g were obtained from Jilin University Animal Center, Jilin province, China. All the experimental mice were kept at the animal care room with the temperature 16–20 °C, the humidity 50%–65% and 12 h light and 12 h dark cycle. The animals were acclimatized to environment for a week with free access to normal commercial diets and water. The diets were purchased from Jilin University Animal Center consisted of crude protein ≥18.0%, crude fat ≥ 4.0% and moisture content ≤ 10.0%,crude ash ≤ 8.0%,crude fiber ≤ 5.0%, calcium 1%–1.8%, as well as phosphor 0.6%–1.2%. All animal experiments were conducted in compliance with “Guide of the care and use of laboratory animals” [29]. 

### 3.6. Induction of T2DM model [MI] 

Streptozotocin (STZ) was used to induce diabetes in this study by being injected to the abdominal cavity of overnight fasted mice at the dose of 160 mg/kg body weight which was freshly dissolved in 0.1 mol/L cold citrate buffer (pH 4.2) [25]. The blood glucose value was evaluated in the next 6th day after the induction. Only mice with a fasting blood glucose concentration above 11.1 mmol/L were considered diabetic and were used in the corresponding groups in the experiment.

### 3.7. Experimental Design

Firstly, the mice were divided into two groups after the acclimation. One was the non-diabetic control group (NC) and another was the diabetic control group (DC). When performing the diabetic induction procedure, mice in the NC group were subjected to citrate buffer, while the DC mice received STZ instead. After the completion of MI, mice both in the NC and DC groups were randomly grouped again with NCs divided into two groups that were non-diabetic control group (NC) and non-diabetic CSFs high dose group (CS) namely, DCs into five groups, including diabetic control group (DC), diabetic dimethylbiguanide group (PC), diabetic CSFs low dose group (LD), diabetic CSFs medium dose group (MD), as well as diabetic CSFs high dose group (HD) and with 10 in each group. During the experimental process, they were assigned to the following treatment, respectively. Group 1 (NC): normal control, non-diabetic control mice administrated tap water only; Group 2 (CS): non-diabetic control mice administrated 500 mg/kg body weight of CSFs; Group 3 (DC): diabetic control mice administrated tap water only; Group 4 (PC): positive control, diabetic control mice administrated 140 mg/kg body weight of dimethylbiguanide; Group 5 (LD): low dose group, diabetic control mice administrated 100 mg/kg body weight of CSFs; Group 6 (MD): medium dose group, diabetic control mice administrated 300 mg/kg body weight of CSFs; Group 7 (HD): high dose group, diabetic control mice administrated 500 mg/kg body weight of CSFs.

All animals were intragastrically administered with the corresponding materials once a day for 28 days. Body weight, water consumption, as well as food intake were determined weekly. Blood samples were obtained by withdrawing from the tails at day 7, day 14, and day 21. At the end of the experiment procedure, mice were deprived from diets overnight and weighed before their sacrifice. Blood samples were collected from the eyes, and centrifuged to obtain the serum which was stored at −20 °C before the further analysis. Livers, kidneys, and pancreas were removed, weighted and stored at −80 °C, respectively. The related organ weight was calculated by the following formula: Related organ weight = (absolute organ weight (g)/final body weight (g)).

### 3.8. Biological Analysis

Serum TC, TG, HDL-C, LDL-C, MDA, and SOD values, in addition to the liver glycogen (LG) level were determined by the prescribed corresponding analytical kits with the detector, ELISA reader (BioTek Instruments, Inc., Winooski, VT, USA). Blood glucose (BG) was measured by the glucose oxidize method. They were expressed as mmol/L with several exceptions that MDA, SOD, and LG value was expressed as umol/mL, NU/mL, and mg/g, respectively.

### 3.9. Statistical Analysis

All the data were expressed as mean ± SD. The significant difference of data in one group was analyzed by unpaired student *t*-test, and statistical differences between different groups were compared by ANOVA followed by Dunnett’s test (SPSS 10.0). *p* value under 0.05 was considered statistically significant. The figures were drawn by Origin 8.5.

## 4. Conclusions

The discovery of the anti-diabetic properties of corn silk, which is abundant and readily accessible to diabetic patients all over the world is attractive with the seriously growing large diabetes population. The data of this investigation showed that the ingestion of CSFs with a dose under 500 mg/kg had no observed adverse effect on normal mice and it had significant anti-diabetic potential, accompanied with anti-oxidant and anti-hyperlipidemic activities. In our present study, we found that after the administration of CSFs for four weeks, the polydipsia, polyphagia, and weight loss symptoms of diabetic mice had been relieved and 500 mg/kg obtained the best effect on preventing diabetic mice from weight loss. The serum BG concentration was ameliorated, and serum TC, TG, LDL-C, MDA and liver glycogen level in the diabetic CSFs high dose group (HD) group were lower than that of diabetic control (DC) group, and furthermore, the HDL-C and SOD value were slightly rising, which implied that CSFs had beneficial anti-diabetic effects by regulating the lipid metabolism and eliminating the oxygen radicals, which protected the organism’s metabolism and repaired the anti-oxidant capacity.
